# Development and application of a prognostic model based on radiomics and artificial intelligence for patients with lung adenocarcinoma brain metastasis

**DOI:** 10.3389/fonc.2026.1864327

**Published:** 2026-06-30

**Authors:** Congying Zheng, Xinyuan Yang, Musen Ye, Kai Tang, Shubin Wang

**Affiliations:** 1 Department of Oncology, Shenzhen Key Laboratory of Gastrointestinal Cancer Translational Research, Cancer Institute, Peking University Shenzhen Hospital, Shenzhen-Peking University-Hong Kong University of Science and Technology Medical Center, Shenzhen, China; 2 Medical College, Shantou University Medical College, Shantou, China; 3 Peking University (PKU)-Shenzhen Clinical Institute of Shantou University Medical College, Shenzhen, China; 4 Intervention and Cell Therapy Center, Peking University Shenzhen Hospital, Shenzhen, China; 5 Department of Radiology, Shenzhen Traditional Chinese Medicine Hospital, The Fourth Clinical Medical College of Guangzhou University of Chinese Medicine, Shenzhen, China; 6 Department of Neurosurgery, Guangdong Provincial People’s Hospital (Guangdong Academy of Medical Sciences), Southern Medical University, Guangzhou, China

**Keywords:** artificial intelligence, clinical characteristics, lung adenocarcinoma brain metastasis, machine learning, prognosis, radiomics

## Abstract

**Introduction:**

Lung cancer with brain metastasis (LCBM) impairs survival in lung adenocarcinoma. High postoperative recurrence rates highlight the necessity of accurate prognostic tools. This study aimed to develop an integrated radiomics-clinical model to improve survival prediction in lung adenocarcinoma patients with brain metastasis.

**Methods:**

The cohort of 176 patients with LCBM was randomly divided into a training set (n=123) and a test set (n=53). The identification of clinical risk factors was performed using both univariate and multivariate logistic regression analyses. A radiomics model was developed based on radiomic features extracted from preoperative magnetic resonance imaging (MRI), following selection with Least Absolute Shrinkage and Selection Operator (LASSO) regression. The performance of the combined nomogram, which integrated significant clinical and radiomic features, was evaluated by the area under the receiver operating characteristic curve (AUC), along with calibration and decision curve analyses.

**Results:**

Multivariate analysis established the EGFR mutation status, number of brain metastases, and Lung-molGPA score as independent prognostic determinants. Performance evaluation of the radiomics model yielded AUCs of 0.862 in the training set and 0.829 in the test set, indicating robust diagnostic performance. The combined nomogram demonstrated superior predictive performance, with AUC values of 0.904 and 0.874 in the training and test sets, respectively, along with good calibration and clinical utility in both cohorts.

**Discussion:**

These findings demonstrate the combined utility of integrating radiomics with clinical parameters to enhance prognostic accuracy, enabling personalized treatment stratification in LCBM and improving clinical decision-making and risk stratification.

## 1 Introduction

Lung cancer was responsible for an estimated 1.9 million deaths globally, following 2.3 million new diagnoses (1). Accounting for 13% of global cancer diagnoses and 18% of cancer mortality, this disease is largely attributable to modifiable risks, primarily tobacco use and environmental exposures (2, 3). In China, lung cancer represents the most frequently diagnosed malignancy and is the primary cause of cancer-associated deaths (4). Despite therapeutic advances, survival rates of patients remain low, particularly metastatic disease (5).

Lung adenocarcinoma brain metastasis (LCBM) remains a devastating complication in oncology, with 20%-65% of lung cancer patients developing intracranial lesions during the disease course (6, 7). These metastases often present with nonspecific neurological symptoms, including headache, and focal deficits (8, 9), yet diagnostic and therapeutic options remain limited. While cranial magnetic resonance imaging (MRI) serves as the principal modalities for diagnosis, the efficacy of therapeutic interventions—including surgery, radiotherapy, and systemic agents—is substantially limited by the blood-brain barrier and profound tumor heterogeneity. Consequently, patient prognosis remains unfavorable (10, 11). Although recent studies have proposed predictive models integrating TNM staging and individualized biomarkers to stratify patients at high risk of BM, critical gaps persist in understanding the pathogenesis, optimizing early detection, and developing targeted therapies (12). Notably, evidence-based management of LCBM requiring craniotomy remains underexplored, underscoring the urgent need for multidisciplinary approaches to improve clinical outcomes.

Radiomics, an emerging discipline bridging medical imaging and quantitative analysis, entails the extraction of minable data from clinical images. These sub-visual features can subsequently be mined to uncover tumor characteristics that are imperceptible through conventional visual assessment (13, 14). By converting images into mineable data, radiomics enables the decoding of tumor heterogeneity and microenvironment, providing a non-invasive approach for assessing disease status and predicting clinical outcomes (15, 16).Its application has recently expanded to include brain metastases, demonstrating significant promise in predicting treatment response, survival outcomes, and disease progression patterns (17–19). Furthermore, the integration of artificial intelligence and radiomics facilitates the combination of imaging data with other information, such as clinical features, to obtain more comprehensive and accurate characterization of brain metastases, thereby enabling the development of robust prognostic models that outperform traditional clinical assessments (20–22).

This study was conducted retrospectively to collect clinical data from patients with LCBM. To first identify clinical predictors of surgical outcomes in LCBM and then develop a prognostic model, we employed a machine learning approach that incorporated both these factors and radiomic features. We aims to contribute a reliable predictive tool for guiding treatment selection in patients with LCBM.

## 2 Methods

### 2.1 Study design and patient selection

This retrospective study was approved by the Ethics Committee of Peking University ShenzhenHospital. Consecutive patients with lung adenocarcinoma brain metastasis (LCBM) who were treated at our institution between January 1, 2015 and May 1, 2025 were screened for eligibility. Patients were included if they had: (1) histopathologically confirmed lung adenocarcinoma; (2) brain metastases confirmed on contrast-enhanced cranial magnetic resonance imaging (MRI) or computed tomography; and (3) complete clinical and follow-up data. Patients were excluded if they lacked histopathological confirmation, had severe hepatic or renal dysfunction, had incomplete clinical records, or had poor-quality MRI scans unsuitable for radiomic analysis. The clinical endpoint was overall survival (OS). For prognostic modeling, patients were dichotomized into a good-prognosis group (OS ≥2 years) and a poor-prognosis group (OS <2 years). Clinical variables collected for analysis included age, sex, smoking history, Karnofsky Performance Status (KPS), EGFR mutation status, metastatic lesion location, extracranial disease status, number of brain metastases, intracranial symptoms, treatment-related variables, Recursive Partitioning Analysis (RPA) class, and Lung-molGPA score. The complete patient enrollment, exclusion, and stratification workflow is presented in the patient selection flowchart (**Supplementary Figure 1**).

### 2.2 Study workflow and cohort allocation

A standardized radiomics workflow was adopted, including patient enrollment, image segmentation, image preprocessing, feature extraction, feature reduction, model construction, and performance evaluation. To enable model development and independent assessment, the dataset was randomly divided into a training cohort and a test cohort at a 7:3 ratio. All feature selection procedures and model construction steps were performed using the training cohort only, whereas the test cohort was held out for independent evaluation to reduce the risk of information leakage and overly optimistic performance estimates. The overall radiomics study workflow is illustrated in **Supplementary Figure 2**.

### 2.3 Tumor segmentation and image preprocessing

Tumor segmentation was manually performed slice by slice using 3D Slicer by an experienced radiologist who was blinded to outcome grouping. Given the heterogeneity of voxel spacing across scanners and acquisition protocols, spatial standardization was performed before feature extraction. Specifically, all images were resampled to isotropic voxels of 1 × 1 × 1 mm3 to improve inter-patient comparability and reduce bias related to acquisition differences. After feature extraction, Z-score normalization was applied to the handcrafted radiomic features to minimize scale-related effects and facilitate downstream model development.

### 2.4 Radiomic feature extraction

**Figure 1:**
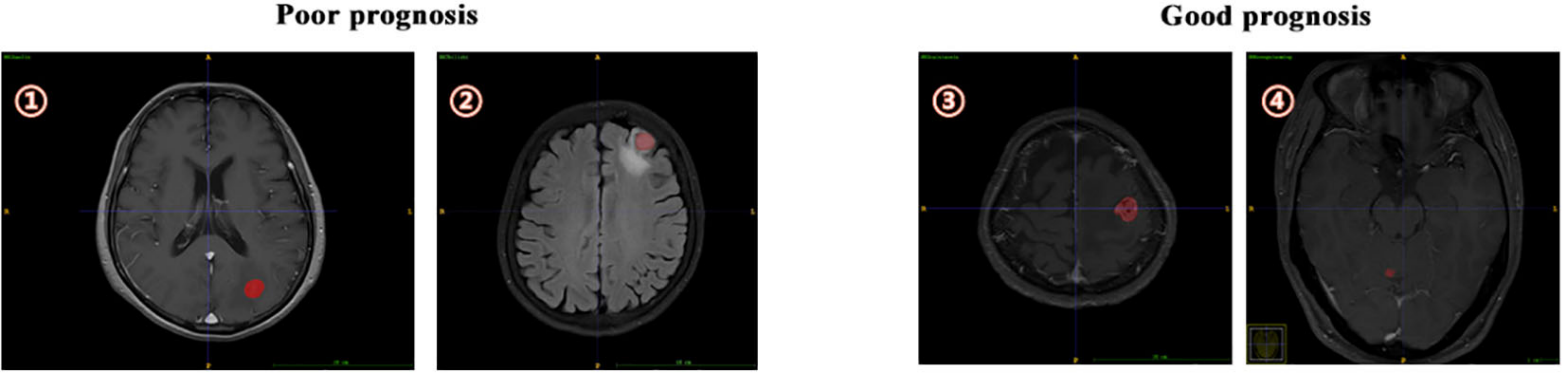
Feature extraction of imageomics.

Radiomic features were extracted from the original MRI data using the PyRadiomics package in Python. The extracted feature set comprised first-order features, shape features, and multiple categories of texture features, including gray-level co-occurrence matrix, gray-level run length matrix, gray-level size zone matrix, gray-level dependence matrix, and neighborhood gray-tone difference matrix features. To capture higher-order spatial information, filtered features were additionally generated by wavelet decomposition (LLL, LLH, LHL, LHH, HLL, HLH, HHL, and HHH) and Laplacian of Gaussian filtering at multiple scales (σ = 2, 3, 4, and 5). This multilevel extraction strategy was used to comprehensively characterize lesion intensity distribution, morphology, and intratumoral heterogeneity. The images of the extracted typical radiomic features in the deceased group and the survival group are shown in **Figure 1**.

### 2.5 Feature reduction and radiomics signature construction

To improve model robustness and limit overfitting, radiomic feature selection was performed in a multistep manner within the training cohort. First, features were prefiltered using the Student’s t-test or Mann-Whitney U test, and only those with P < 0.05 were retained. Second, feature redundancy was reduced by pairwise Spearman correlation analysis; when two features showed a correlation coefficient >0.90, only one was retained. Third, the remaining features were entered into least absolute shrinkage and selection operator (LASSO) regression, and the optimal regularization parameter λ was selected using 10-fold cross-validation according to the minimum criterion. Features with nonzero coefficients were finally retained to construct the radiomics signature. This hierarchical reduction strategy was intended to balance dimensionality reduction, feature stability, and predictive relevance.

### 2.6 Clinical model and combined model development

Clinical predictors were first assessed by univariable logistic regression in the training cohort. Variables showing prognostic relevance were then entered into a multivariable logistic regression model to identify independent clinical predictors. On this basis, a clinical model was established. In parallel, a radiomics model was developed from the selected radiomics signature. To assess the incremental value of radiomics beyond conventional clinical factors, a combined model integrating the radiomics signature and the independent clinical predictors was subsequently constructed. A nomogram was further generated from the combined model to facilitate individualized risk estimation in clinical practice.

### 2.7 Model performance evaluation

Model discrimination was evaluated in both the training and test cohorts using the area under the receiver operating characteristic curve (AUC), accuracy, sensitivity, specificity, positive predictive value (PPV), and negative predictive value (NPV). Model calibration was assessed using calibration curves and the Hosmer-Lemeshow goodness-of-fit test. The clinical usefulness of each model was further explored by decision curve analysis (DCA). In addition, confusion matrices were used to visualize classification performance, and receiver operating characteristic curves were compared using the DeLong test where appropriate. This multidimensional evaluation framework was used to comprehensively assess discrimination, calibration, and potential clinical utility.

### 2.8 Statistical analysis

Statistical analyses were performed using the statsmodels package in Python (version 0.13.2). Continuous variables were compared using the Student’s t-test or Mann-Whitney U test, as appropriate, whereas categorical variables were compared using the chi-square test or Fisher’s exact test. Odds ratios (ORs) and 95% confidence intervals (CIs) were calculated for logistic regression analyses. All statistical tests were two-sided, and P < 0.05 was considered statistically significant.

## 3 Results

### 3.1 Patient characteristics and cohort comparability

A total of 176 patients with LCBM were enrolled, including 123 patients in the training cohort and 53 in the test cohort. Baseline clinical characteristics were generally well balanced between the two cohorts, with no significant differences in age, KPS, sex, smoking history, EGFR mutation status, number of brain metastases, metastatic lesion location, extracranial metastasis, intracranial symptoms, treatment-related variables, RPA class, or Lung-molGPA score. These findings indicated that the random split yielded two comparable cohorts suitable for model development and independent validation (**Table 1**).

**Table 1:** Comparison of clinical baseline data between training set and test set.

Clinical features	All (n=176)	Test set (n=53)	Training set (n=123)	P
Age	57.898 ± 11.051	56.755 ± 10.586	58.390 ± 11.252	0.369
KPS	77.784 ± 16.219	79.434 ± 13.216	77.073 ± 17.355	0.664
Gender				0.845
female	96 (54.545)	30 (56.604)	66 (53.659)	
male	80 (45.455)	23 (43.396)	57 (46.341)	
Smoking history				0.438
No	138 (78.409)	44 (83.019)	94 (76.423)	
Yes	38 (21.591)	9 (16.981)	29 (23.577)	
EGFR mutations				1.0
No	40 (22.727)	12 (22.642)	28 (22.764)	
Yes	136 (77.273)	41 (77.358)	95 (77.236)	
Number of brain metastases				0.601
1~3	53 (30.114)	14 (26.415)	39 (31.707)	
≥3	123 (69.886)	39 (73.585)	84 (68.293)	
Metastatic lesion location				0.550
Supratentorial/infratentorial cerebellum	161 (91.477)	50 (94.340)	111 (90.244)	
Infratentorial cerebellum only	15 (8.523)	3 (5.660)	12 (9.756)	
Extracranial metastasis				0.346
No	19 (10.795)	8 (15.094)	11 (8.943)	
Yes	157 (89.205)	45 (84.906)	112 (91.057)	
Intracranial symptoms				0.408
No	103 (58.523)	34 (64.151)	69 (56.098)	
Yes	73 (41.477)	19 (35.849)	54 (43.902)	
Targeted therapy				0.742
No	7 (3.977)	3 (5.660)	4 (3.252)	
Yes	169 (96.023)	50 (94.340)	119 (96.748)	
Radiotherapy				0.0876
No	63 (35.795)	26 (49.057)	37 (30.081)	
Yes	113 (64.205)	27 (50.943)	86 (69.919)	
Chemotherapy				0.866
No	149 (84.659)	44 (83.019)	105 (85.366)	
Yes	27 (15.341)	9 (16.981)	18 (14.634)	
RPA				0.79
1	10 (5.682)	4 (7.547)	6 (4.878)	
2	75 (42.614)	23 (43.396)	52 (42.276)	
3	91 (51.705)	26 (49.057)	65 (52.846)	
Lung-molGPA				1.0
<3	85 (48.295)	26 (49.057)	59 (47.967)	
≥3	91 (51.705)	27 (50.943)	64 (52.033)	

### 3.2 Clinical characteristics associated with prognosis in the training cohort

Within the training cohort, 36 patients were assigned to the good-prognosis group and 87 to the poor-prognosis group. Univariable analysis showed that EGFR mutation status, number of brain metastases, and Lung-molGPA score differed significantly between the two outcome groups. Specifically, the poor-prognosis group had a substantially higher frequency of EGFR mutation, a higher proportion of patients with at least three brain metastases, and a higher proportion of patients with Lung-molGPA scores <3. No statistically significant between-group differences were observed for age, KPS, sex, smoking history, metastatic lesion location, extracranial metastasis, intracranial symptoms, targeted therapy, radiotherapy, chemotherapy, or RPA class (**Table 2**).

**Table 2:** Univariate analysis of training set clinical baseline data.

Clinical features	All (n=123)	Good prognosis (n=36)	Poor prognosis (n=87)	P
Age	58.390 ± 11.252	56.917 ± 11.380	59.000 ± 11.207	0.352
KPS	77.073 ± 17.355	80.278 ± 11.585	75.747 ± 19.146	0.415
Gender				0.386
female	66 (53.659)	22 (61.111)	44 (50.575)	
male	57 (46.341)	14 (38.889)	43 (49.425)	
Smoking history				0.353
No	94 (76.423)	30 (83.333)	64 (73.563)	
Yes	29 (23.577)	6 (16.667)	23 (26.437)	
EGFR mutations				<0.001
No	28 (22.764)	21 (58.333)	7 (8.046)	
Yes	95 (77.236)	15 (41.667)	80 (91.954)	
Number of brain metastases				0.01
1~3	39 (31.707)	18 (50.000)	21 (24.138)	
≥3	84 (68.293)	18 (50.000)	66 (75.862)	
Metastatic lesion location				0.509
Supratentorial/infratentorial cerebellum	111 (90.244)	31 (86.111)	80 (91.954)	
Infratentorial cerebellum only	12 (9.756)	5 (13.889)	7 (8.046)	
Extracranial metastasis				0.846
No	11 (8.943)	4 (11.111)	7 (8.046)	
Yes	112 (91.057)	32 (88.889)	80 (91.954)	
Intracranial symptoms				1.0
No	69 (56.098)	20 (55.556)	49 (56.322)	
Yes	54 (43.902)	16 (44.444)	38 (43.678)	
Targeted therapy				1.0
No	4 (3.252)	1 (2.778)	3 (3.448)	
Yes	119 (96.748)	35 (97.222)	84 (96.552)	
Radiotherapy				0.150
No	37 (30.081)	7 (19.444)	30 (34.483)	
Yes	86 (69.919)	29 (80.556)	57 (65.517)	
Chemotherapy				0.490
No	105 (85.366)	29 (80.556)	76 (87.356)	
Yes	18 (14.634)	7 (19.444)	11 (12.644)	
RPA				0.724
1	6 (4.878)	2 (5.556)	4 (4.598)	
2	52 (42.276)	17 (47.222)	35 (40.230)	
3	65 (52.846)	17 (47.222)	48 (55.172)	
Lung-molGPA				0.001
<3	64 (52.033)	10 (27.778)	54 (62.069)	
≥3	59 (47.967)	26 (72.222)	33 (37.931)	

### 3.3 Independent clinical predictors of poor prognosis

Variables identified in the training cohort were further analyzed using multivariable logistic regression. The number of brain metastases (OR = 1.176, 95% CI: 1.036-1.336, P = 0.036), Lung-molGPA score (OR = 0.831, 95% CI: 0.737-0.937, P = 0.012), and EGFR mutation status (OR = 1.618, 95% CI: 1.398-1.874, P < 0.001) remained independently associated with prognosis. These results indicated that a greater intracranial metastatic burden and EGFR mutation were associated with poorer outcome, whereas a higher Lung-molGPA score was associated with more favorable prognosis (**Table 3**).

**Table 3:** Univariate and multivariate logistic regression analysis of clinical data in the training set.

Variable	OR univariate	95%CI lower limit	95%CI upper limit	P-value
Radiotherapy	0.862	0.744	1.000	0.100
Location	0.872	0.692	1.097	0.324
Chemotherapy	0.893	0.736	1.084	0.336
Targeted_therapy	0.957	0.650	1.408	0.850
Symptom	0.994	0.865	1.141	0.939
KPS	0.997	0.993	1.001	0.189
Age	1.003	0.997	1.010	0.352
RPA	1.055	0.939	1.184	0.450
Extracranial_metastasis	1.081	0.850	1.374	0.591
Gender	1.092	0.952	1.252	0.290
Smoking	1.119	0.953	1.314	0.249
Number	1.281	1.111	1.477	0.005
Lung_molGPA	0.752	0.660	0.875	0.000
EGFR	1.808	1.576	2.073	0.000
	OR multivariate	95%CI lower limit	95%CI upper limit	P-value
Number	1.176	1.036	1.336	0.036
Lung_molGPA	0.831	0.737	0.937	0.012
EGFR	1.618	1.398	1.874	0.0

### 3.4 Radiomic feature reduction and radiomics signature development

**Figure 2:**
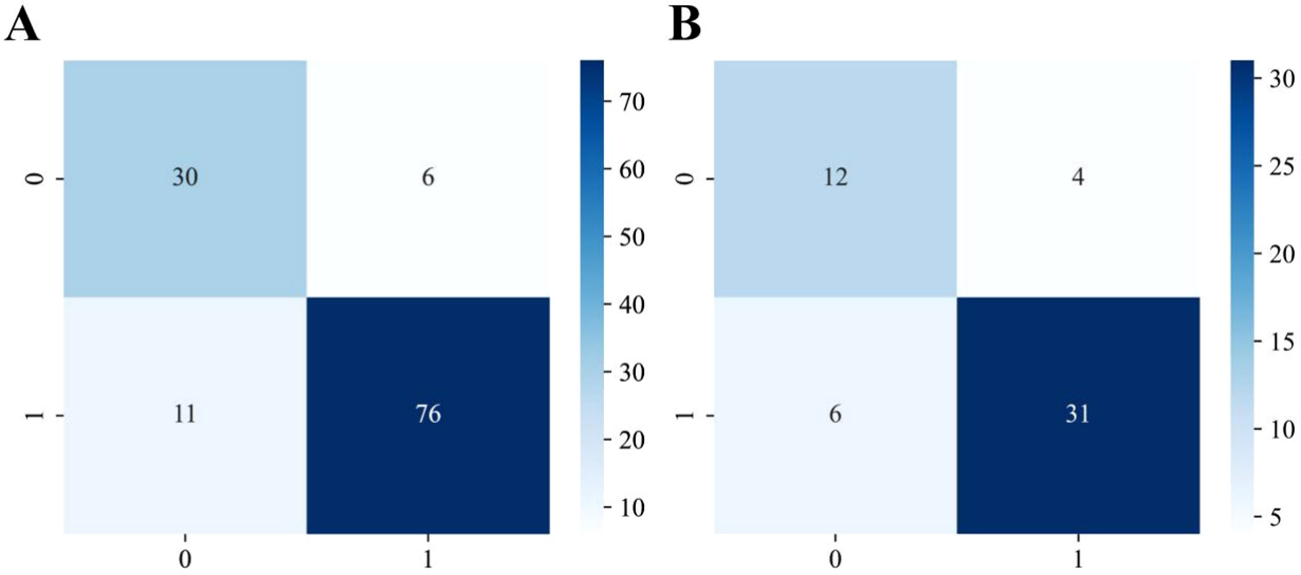
Radiomics feature extraction. **(A)** 10 fold cross validation coefficient. **(B)** 10 fold cross validated MSE. **(C)** non zero coefficient features after feature selection using minimum absolute shrinkage and selection operator regression.

A total of 1,197 radiomic features were extracted from MRI, including 234 first-order features, 14 shape features, and 949 texture features. After initial univariable filtering, 78 candidate features were retained. Redundancy reduction by correlation analysis further reduced the feature set to 33 features. These features were then subjected to LASSO regression with 10-fold cross-validation, and eight features with nonzero coefficients were ultimately retained to construct the radiomics signature (**Table 4**; **Figure 2**). This sequential selection process substantially reduced feature dimensionality while preserving the most informative prognostic descriptors.

**Table 4:** Characteristic categories.

No.	Feature category	Feature count
1	firstorder	234
0	glcm	286
4	gldm	182
2	glrlm	208
3	glszm	208
5	ngtdm	65
6	shape	14

### 3.5 Performance of the radiomics model

The radiomics model demonstrated good discrimination in both cohorts. In the training cohort, it achieved an AUC of 0.862 (95% CI: 0.795-0.927), with an accuracy of 0.764, sensitivity of 0.724, specificity of 0.861, PPV of 0.926, and NPV of 0.564. In the test cohort, the radiomics model yielded an AUC of 0.829 (95% CI: 0.717-0.941), with an accuracy of 0.736, sensitivity of 0.703, specificity of 0.812, PPV of 0.897, and NPV of 0.542. These findings indicated that the radiomics signature alone provided stable and clinically meaningful prognostic stratification across both development and held-out cohorts (**Table 5**).

**Table 5:** Radiomics machine learning modeling analysis.

Cohort	Model	AUC	AUC 95%CI	Acc	Sen	Spe	PPV	NPV
Train	Radiomics	0.862	0.795 - 0.927	0.764	0.724	0.861	0.926	0.564
Test	Radiomics	0.829	0.717 - 0.941	0.736	0.703	0.812	0.897	0.542

### 3.6 Comparative performance of the clinical, radiomics, and combined models

The clinical model showed moderate prognostic performance, with AUCs of 0.770 (95% CI: 0.676-0.863) in the training cohort and 0.740 (95% CI: 0.580-0.899) in the test cohort. The radiomics model outperformed the clinical model in both cohorts. After integrating the radiomics signature with the independent clinical predictors, the combined model achieved the best overall performance. Its AUC reached 0.904 (95% CI: 0.845-0.962) in the training cohort and 0.874 (95% CI: 0.773-0.974) in the test cohort. Corresponding accuracy values were 0.862 and 0.811, sensitivity values were 0.874 and 0.838, specificity values were 0.833 and 0.750, PPV values were 0.927 and 0.886, and NPV values were 0.732 and 0.667, respectively. Overall, these results demonstrated that integrating radiomic and clinical information improved prognostic discrimination beyond either component alone (**Table 6**).

**Table 6:** Radiomics combined with clinical risk factor modeling analysis.

Cohort	Model	AUC	AUC 95%CI	Acc	Sen	Spe	PPV	NPV
Train	Clinical	0.770	0.676-0.863	0.772	0.851	0.583	0.831	0.618
Test	Clinical	0.740	0.580-0.899	0.698	0.649	0.812	0.889	0.500
Train	Radiomics	0.862	0.795-0.927	0.764	0.724	0.861	0.926	0.564
Test	Radiomics	0.829	0.717-0.941	0.736	0.703	0.812	0.897	0.542
Train	Combined	0.904	0.845-0.962	0.862	0.874	0.833	0.927	0.732
Test	Combined	0.874	0.773-0.974	0.811	0.838	0.750	0.886	0.667

### 3.7 Calibration and clinical utility of the combined model

**Figure 3:**
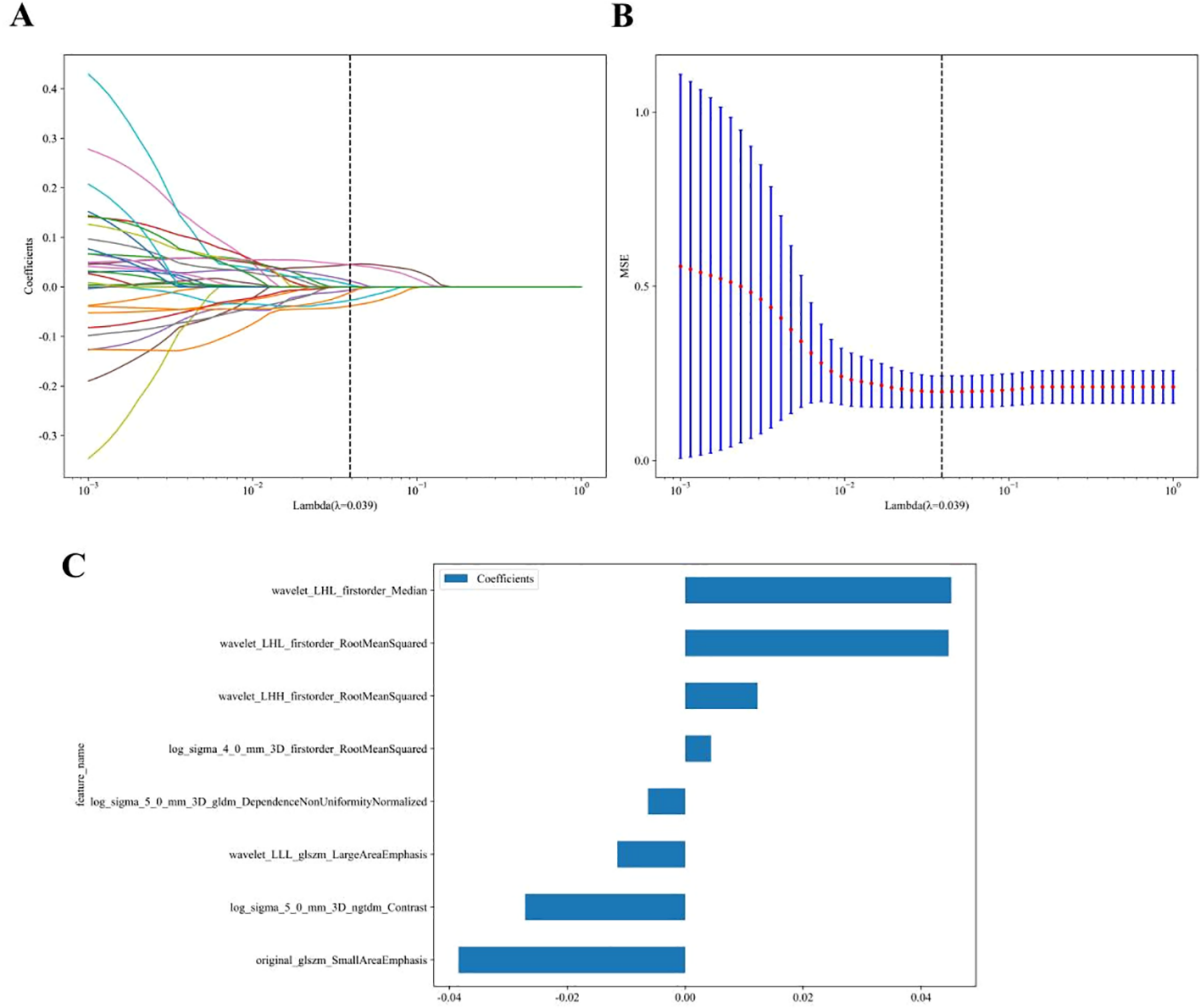
Calibration curves and DCA curves for the clinical, radiological, and combined models. **(A)** calibration curve for training set. **(B)** calibration curve for test set. **(C)** DCA curve for training set. **(D)** DCA curve for test set.

**Figure 4:**
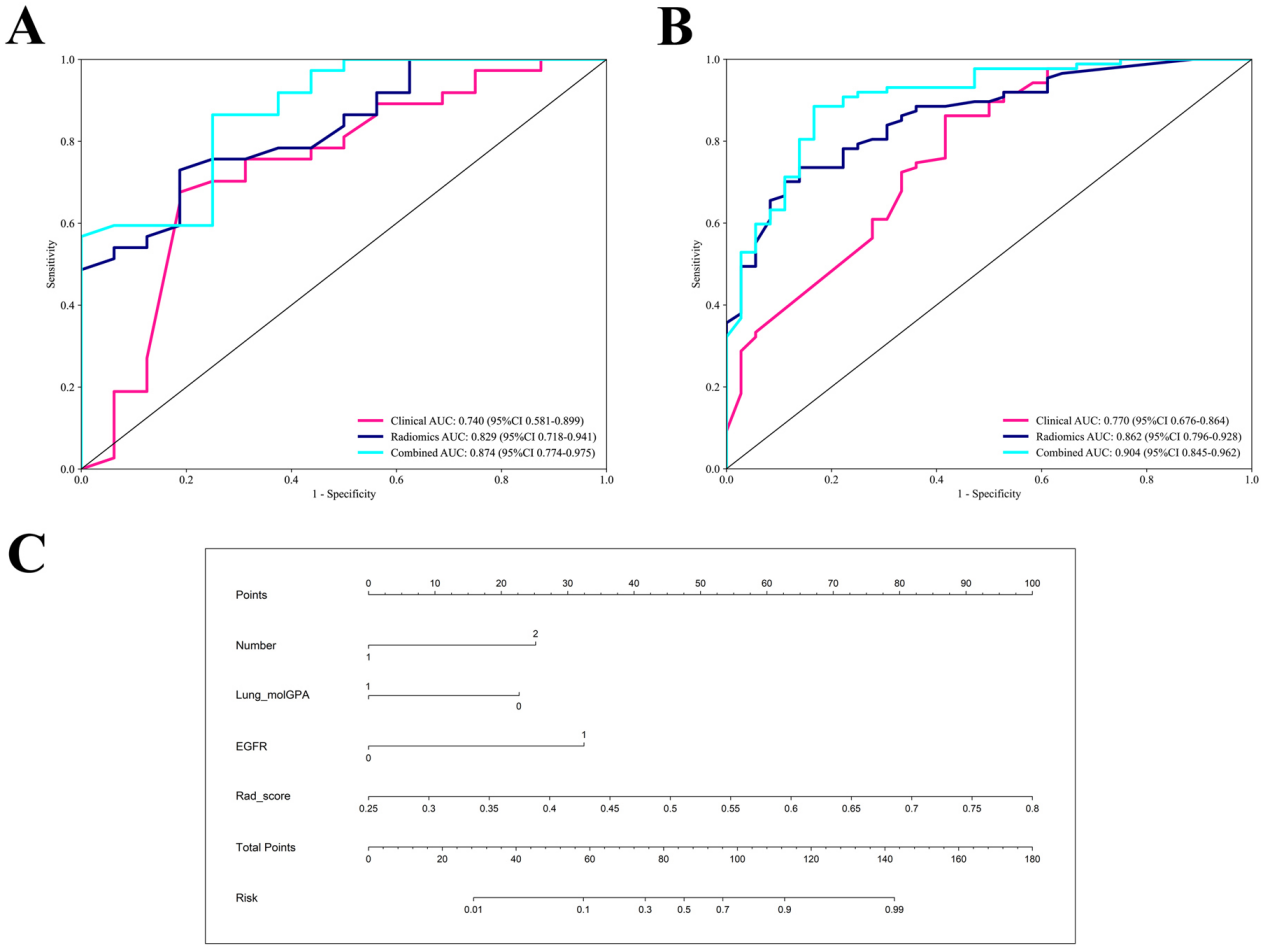
ROC curves and nomograms for the clinical, radiological, and combined models. **(A)** ROC curve for training set. **(B)** ROC curve for test set. **(C)** nomogram prediction model.

**Figure 5:**
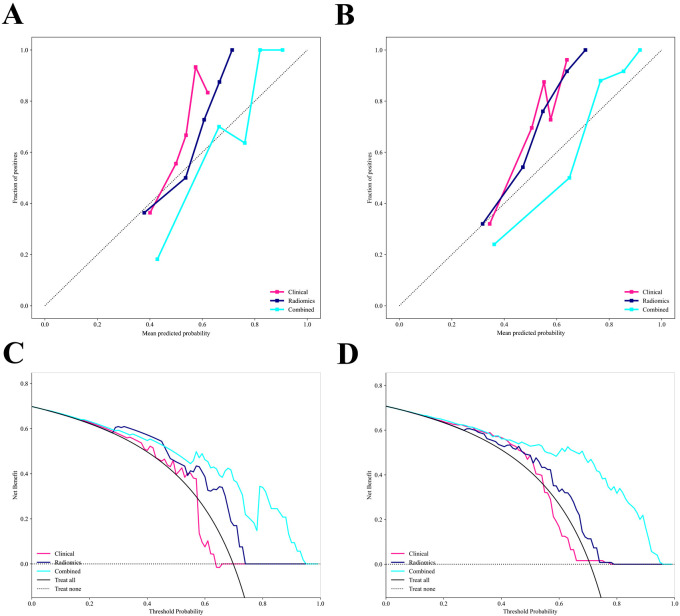
Confusion matrix of the combined model. **(A)** confusion matrix of the training set. **(B)** confusion matrix of the test set.

Calibration analysis showed acceptable goodness of fit for all three models in both cohorts, with all Hosmer-Lemeshow P values exceeding 0.05. For the combined model, the Hosmer-Lemeshow test yielded P = 0.163 in the training cohort and P = 0.155 in the test cohort, indicating good agreement between predicted and observed outcomes. Calibration curves further supported model stability, while DCA demonstrated superior net clinical benefit of the combined model over a range of threshold probabilities. In addition, the confusion matrices in the training and test cohorts visually confirmed the favorable classification performance of the combined model. A nomogram derived from the combined model was constructed to enable intuitive and individualized prognostic estimation (**Table 7**; **Figures 3**–**5**).

**Table 7:** Hosmer_lemeshow_test.

Cohort	Clinical	Radiomics	Combined	Cohort
P	0.052	0.055	0.163	Train
P	0.222	0.368	0.155	Test

## 4 Discussion

Lung cancer frequently involves BM. Although therapies such as radiotherapy, chemotherapy, and targeted treatment have achieved certain success in managing LCBM, the risk of postoperative recurrence remains high, significantly impacting patient survival (23). This retrospective study included 176 patients with LCBM, of whom 52 had good outcomes and 124 had poor outcomes, dying within two years after surgery. Therefore, identifying key factors influencing recurrence and enabling early intervention is crucial for improving prognosis and reducing mortality in patients with LCBM.

Machine learning is increasingly leveraged in medical research to analyze complex datasets, identify patterns, and support clinical decision-making (24). Therefore, we employed a machine learning approach to prognosticate outcomes in patients with LCBM. The study population was composed of training and test sets, totaling 176 patients. Univariate analysis of clinical characteristics between the favorable and poor prognosis groups within the training set revealed that EGFR mutations, number of brain metastases, and Lung-molGPA score were risk factors associated with poor prognosis Multivariate logistic regression further confirmed that these factors were independently associated with an unfavorable prognosis in LCBM.

In NSCLC, particularly lung adenocarcinoma, EGFR mutations are one of the most characterized oncogenic drivers (25). Notably, EGFR mutation is a risk factor for brain metastasis in NSCLC (7). In LCBM, Shin et al. (26) previously reported a statistically higher frequency of EGFR mutations in patients with BM (64.7% with BM vs. 39.8% without metastasis and 40.2% with extracranial metastases; p=0.005). Further analysis of 133 patients who underwent surgical resection demonstrated that, after adjusting for pathological N stage, EGFR mutation status remained an independent risk factor for BM. Moreover, Kim et al. (27) evaluated the incidence of BM in 1495 patients with NSCLC at 6, 12, 18, and 24 months after initial assessment. They found that the incidence rates were higher in adenocarcinoma patients with EGFR mutations (0.7%, 2.5%, 6.3%, and 12.3% respectively) than in those without EGFR mutations (0.4%, 1.8%, 2.9%, and 4.4%) (p<0.001). In a cohort of 1,109 lung adenocarcinoma patients, EGFR mutation-positive status was found to be an independent predictor of BM (hazard ratio, 1.78; p=0.04). While EGFR mutations are well-established risk factors for BM development in NSCLC, their prognostic role in patients who already have established LCBM remains less defined. In this study, we specifically investigated whether EGFR mutation status influences postoperative survival in LCBM patients. Our analysis revealed that the proportion of EGFR mutations was markedly higher in the poor-prognosis group (91.954%) than in the favorable-prognosis group (41.667%) within the training cohort. Multivariate analysis further confirmed EGFR mutation as an independent risk factor for unfavorable prognosis in LCBM patients (OR = 1.618, 95% CI: 1.398-1.874, P < 0.001). This finding extends prior knowledge by demonstrating that beyond predicting BM occurrence, EGFR mutation status also predicts poor outcomes after craniotomy in patients with established LCBM. Therefore, incorporating molecular markers such as EGFR into postoperative prognostic evaluations for LCBM is necessary.

Moreover, our study identified the number of brain metastases as an independent risk factor for poor prognosis (OR = 1.176), with the poor prognosis group in the training set showing a higher proportion of patients with more than three brain metastases. This results may indicate that multiple intracranial lesions reflect more aggressive tumor behavior, including enhanced metastatic potential and invasiveness, which may facilitate rapid intracranial progression.

The Lung-molGPA is a specialized prognostic scoring system designed for patients with LCBM. It integrates molecular biomarkers with traditional clinical variables to stratify patients into distinct risk groups with differential survival outcomes (28). (29) found that for patients with LCBM, the median survival times for Lung-molGPA scores of 0-1, 1.5-2, 2.5-3, and 3.5–4 were 25 months, 30 months, 35 months, and over 4 years, respectively, which showed a significant difference from the results reported by (30) (4.27, 6.96, 14.68, and 18.89 months, p < 0.0001). Our analysis validated the Lung-molGPA as an independent prognostic determinant among LCBM patients. A score of < 3 was more prevalent among patients in the poor prognosis group of the training set. Thus, these consistent results strongly support the prognostic utility of the Lung-molGPA for LCBM. The concentration of patients with lower scores (<3) in the poor prognosis group within our cohort further validates its utility for risk stratification in clinical practice.

Furthermore, a radiomic model developed from extracted features in LCBM patients showed good predictive performance in our analysis, which was validated by AUCs of 0.862 and 0.829 in the training and test sets. Machine learning-based radiomic prognostic models have shown favorable predictive efficacy in cancers (31, 32). We integrated radiomic and clinical features to develop a combined model in our study. This model demonstrated superior performance to either model alone, achieving AUCs of 0.904 and 0.874 in the training and test sets, respectively. These findings highlight the synergistic advantage of integrating radiomics with clinical features in improving prognostic accuracy for LCBM, validating the potential of multi-modal models to provide more reliable evidence for individualized clinical decision-making.

Several limitations of this study should be acknowledged. First, comprehensive next−generation sequencing was not uniformly available for all patients during the study period (2015–2025). While EGFR mutation status was routinely tested as part of standard clinical practice, testing for other driver alterations (e.g., ALK, ROS1, KRAS, BRAF) was performed only in a subset of patients, leading to a substantial proportion of missing data. Consequently, these other mutations could not be included in our prognostic model. Second, the retrospective single−center design may introduce selection bias, and the sample size, although acceptable, is relatively modest. Third, external validation in an independent multi−institutional cohort is warranted to confirm the generalizability of our combined radiomics−clinical nomogram. Future prospective studies with systematic molecular profiling are needed to further refine the prognostic model.

In conclusion, the integration of clinical factors and multiparametric MRI-based radiomics into a nomogram enables the visual prediction of prognosis for patients with LCBM. Moreover, its predictive performance is superior to that of the individual radiomics model or clinical model, thereby serving as a more effective clinical auxiliary tool for the assessment of prognosis in patients with LCBM. Currently, this model is established and validated exclusively in patients who have already developed brain metastasis. In future research, we will further enroll lung adenocarcinoma patients without brain metastasis to verify and optimize this analytical system. It is promising to transform the present model into a predictive tool for identifying individuals at high risk of developing brain metastasis, rather than merely conducting prognostic evaluation for patients with established intracranial lesions. This extended application will facilitate early risk stratification and individualized preventive intervention in clinical practice.

## Data Availability

The raw data supporting the conclusions of this article will be made available by the authors, without undue reservation.

## Supplementary material

Supplementary Figure 1 The patient selection flowchart.

Supplementary Figure 2 Study workflow diagram.
