# Supplementary figures and images for: Development and application of a prognostic model based on radiomics and artificial intelligence for patients with lung adenocarcinoma brain metastasis
Source: Front Oncol. 2026 Jun 30;16:1864327. doi: 10.3389/fonc.2026.1864327 (PMC13364902; doi:10.3389/fonc.2026.1864327)

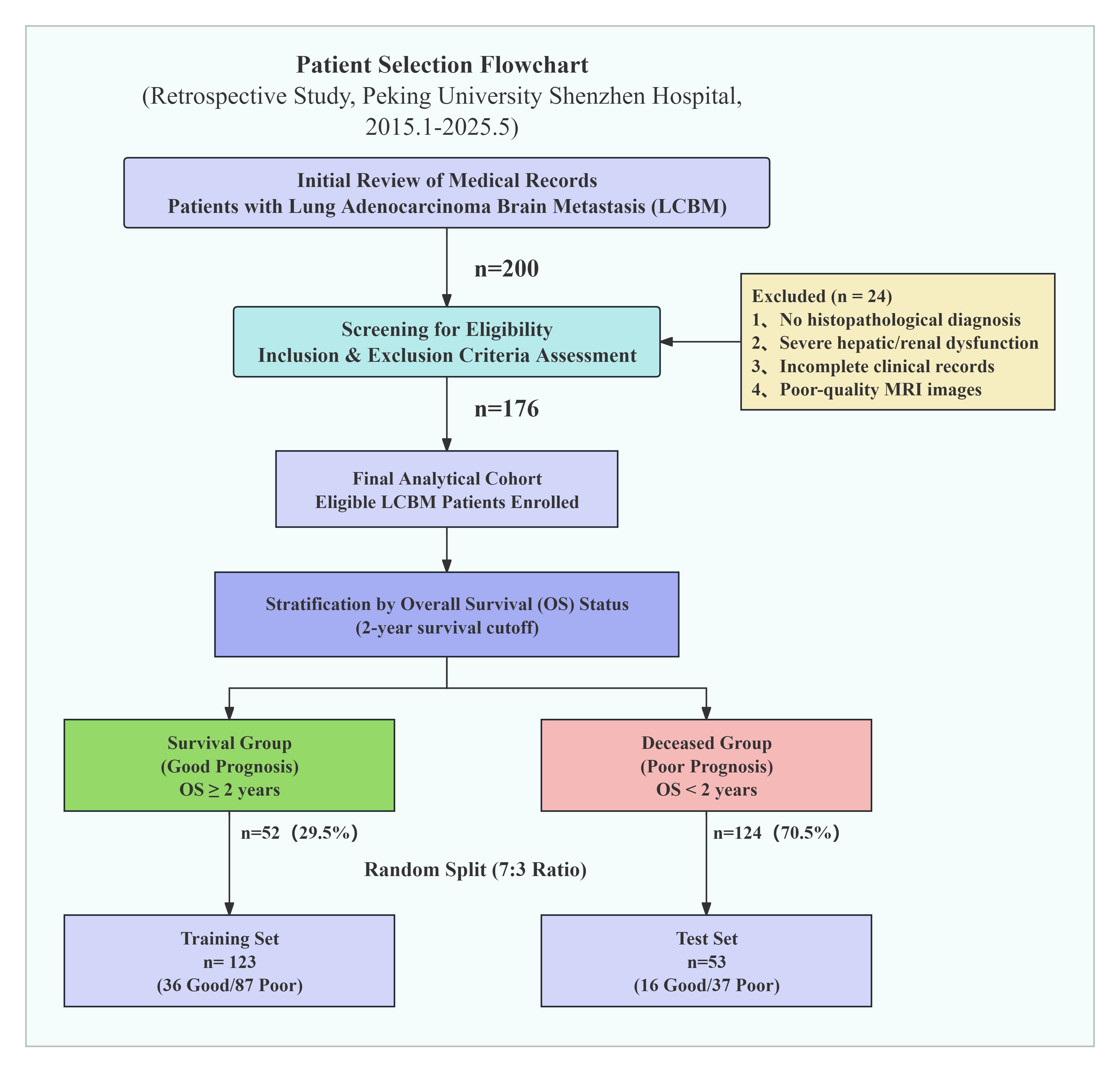

Supplement: Supplementary Figure 1 — The patient selection flowchart. [file Image1.jpeg]

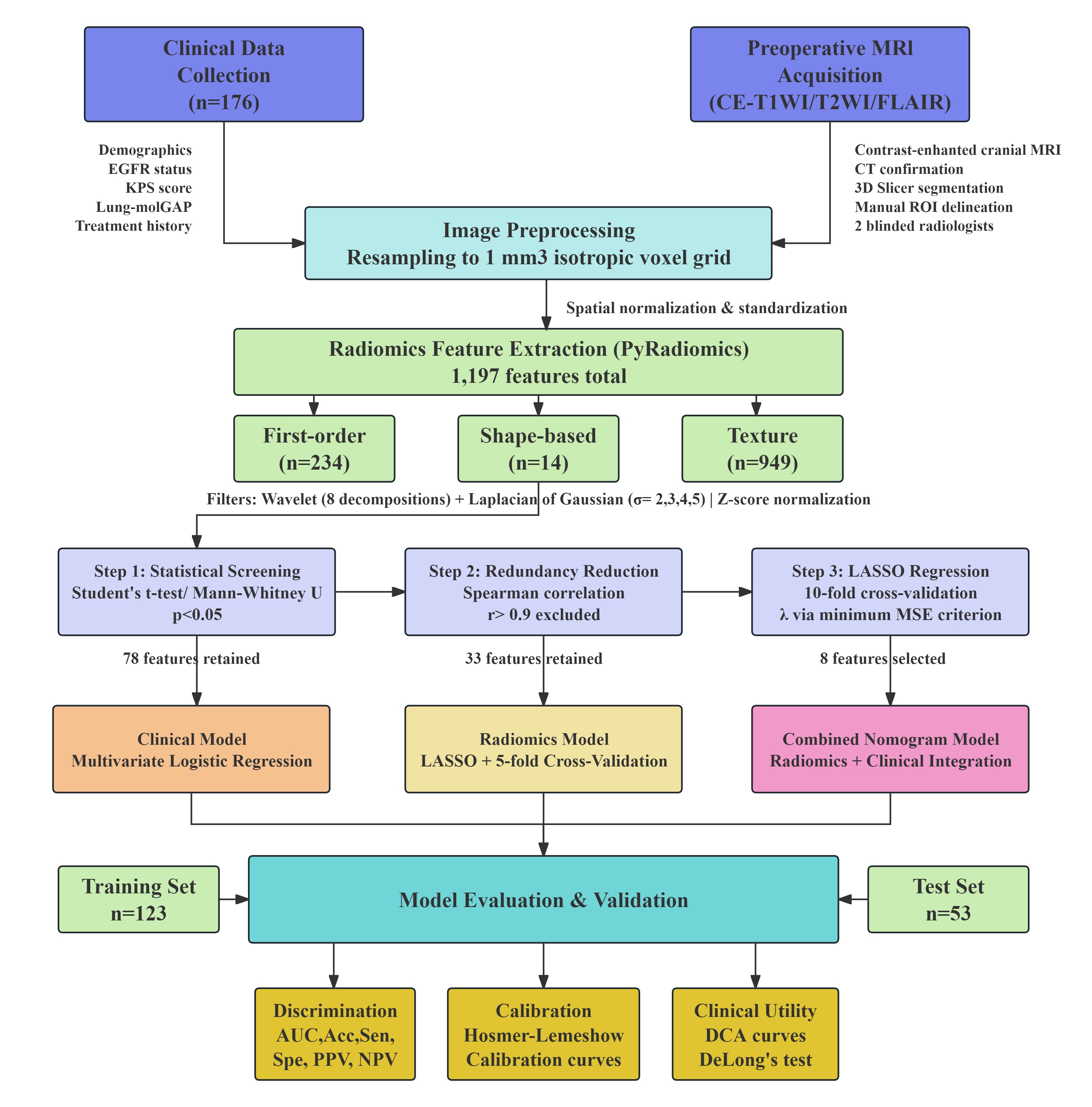

Supplement: Supplementary Figure 2 — Study workflow diagram. [file Image2.jpeg]
